# Use and effectiveness of pegfilgrastim prophylaxis in US clinical practice:a retrospective observational study

**DOI:** 10.1186/s12885-019-6010-9

**Published:** 2019-08-09

**Authors:** Derek Weycker, Robin Doroff, Ahuva Hanau, Charles Bowers, Rajesh Belani, David Chandler, Alexander Lonshteyn, Mark Bensink, Gary H. Lyman

**Affiliations:** 1 Policy Analysis Inc. (PAI), Four Davis Court, Brookline, MA 02445 USA; 2 Amgen Inc., Thousand Oaks, CA USA; 3 Fred Hutchinson Cancer Research Center and the University of Washington, Seattle, WA USA

**Keywords:** Febrile neutropenia, Pegfilgrastim, Neulasta, Granulocyte colony-stimulating factor

## Abstract

**Background:**

Febrile neutropenia (FN) is a serious complication of myelosuppressive chemotherapy. Clinical practice guidelines recommend routine prophylactic coverage with granulocyte colony-stimulating factor (G-CSF)—such as pegfilgrastim—for most patients receiving chemotherapy with an intermediate to high risk for FN. Patterns of pegfilgrastim prophylaxis during the chemotherapy course and associated FN risks in US clinical practice have not been well characterized.

**Methods:**

A retrospective cohort design and data from two commercial healthcare claims repositories (01/2010–03/2016) and Medicare Claims Research Identifiable Files (01/2007–09/2015) were employed. Study population included patients who had non-metastatic breast cancer or non-Hodgkin’s lymphoma and received intermediate/high-risk regimens. Pegfilgrastim prophylaxis use and FN incidence were ascertained in each chemotherapy cycle, and all cycles were pooled for analyses. Adjusted odds ratios for FN were estimated for patients who did versus did not receive pegfilgrastim prophylaxis in that cycle.

**Results:**

Study population included 50,778 commercial patients who received 190,622 cycles of chemotherapy and 71,037 Medicare patients who received 271,944 cycles. In cycle 1, 33% of commercial patients and 28% of Medicare patients did not receive pegfilgrastim prophylaxis, and adjusted odds of FN were 2.6 (95% CI 2.3–2.8) and 1.6 (1.5–1.7), respectively, versus those who received pegfilgrastim prophylaxis. In cycle 2, 28% (commercial) and 26% (Medicare) did not receive pegfilgrastim prophylaxis; corresponding adjusted FN odds were comparably elevated (1.9 [1.6–2.2] and 1.6 [1.5–1.8]). Results in subsequent cycles were similar. Across all cycles, 15% of commercial patients and 23% of Medicare patients did not receive pegfilgrastim prophylaxis despite having FN in a prior cycle, and prior FN increased odds of subsequent FN by 2.1–2.4 times.

**Conclusions:**

Notwithstanding clinical practice guidelines, a large minority of patients did not receive G-CSF prophylaxis, and FN incidence was substantially higher among this subset of the population. Appropriate use of pegfilgrastim prophylaxis may reduce patient exposure to this potentially fatal but largely preventable complication of myelosuppressive chemotherapy.

**Electronic supplementary material:**

The online version of this article (10.1186/s12885-019-6010-9) contains supplementary material, which is available to authorized users.

## Background

Low neutrophil count (“neutropenia”) is a frequent side effect of myelotoxic chemotherapy and increases infection risk. Neutropenia in the presence of fever (“febrile neutropenia” [FN]) typically necessitates inpatient care and may result in delays, reductions, and/or discontinuation of chemotherapy that can—in turn—lead to adverse outcomes [1–11]. For patients whose projected FN risk is high (> 20%) based on the planned chemotherapy regimen and individual risk factors (e.g., age > 65 years, comorbidity profile), prophylaxis with granulocyte colony-stimulating factor (G-CSF) is recommended [1, 9, 12–14]. However, published evidence indicates that many patients who are candidates for G-CSF are not administered it, or are not administered it per recommendations, and thus may be at elevated risk of FN and hospitalization [12–27].

Pegfilgrastim, which requires only a single dose per cycle of chemotherapy, is the most commonly used CSF agent in the US, with previous evaluations reporting that it accounted for > 90% of all CSF prophylaxis use in their study populations [12, 15, 20, 24, 28, 29]. Moreover, pegfilgrastim has been reported—in post-hoc analyses of clinical trials, meta-analyses of clinical trials, and real-world evaluations—to be more efficacious and effective than other CSFs in preventing FN [12, 15, 20, 28–33]. Notwithstanding the availability of pegfilgrastim since 2002 and clinical practice guidelines supporting its use, relatively little is known about patterns of pegfilgrastim use across multiple cycles of chemotherapy during the course, the influence of FN on subsequent pegfilgrastim use, and the impact of pegfilgrastim on the incidence of FN [34]. We therefore undertook two retrospective observational cohort studies, the first using data from two large healthcare claims repositories and the second using data from Medicare Claims Research Identifiable Files (RIFs), to examine these issues among patients with non-metastatic breast cancer or non-Hodgkin’s lymphoma (NHL) receiving chemotherapy regimens with an intermediate to high risk for FN in US clinical practice.

## Methods

The methods of this study—including the design, identification of source/study populations, and variable definitions—are largely the same as those employed in prior evaluations conducted by some of the investigators involved in the present research [16, 17, 23, 35]. An additional file provides a detailed description of study methods and source databases (Additional file 1); a brief description follows.

### Study design and data source

A retrospective observational cohort design was employed to analyze patient-level data from commercial claims and Medicare claims, respectively. For commercial claims (January 2010 – March 2016), data were obtained from two repositories: the Truven Health Analytics MarketScan® Commercial Claims and Encounters and Medicare Supplemental and Coordination of Benefits Databases (MarketScan Database) and the IMS LifeLink™ PharMetrics Plus Health Plan Claims Database (PharMetrics Plus Database). For Medicare claims (January 2007 – September 2015), data were obtained from the RIFs of the Centers for Medicare and Medicaid Services (CMS). Data extracts were de-identified prior to their release to study investigators and thus their use for health services research is compliant with the Health Insurance Portability and Accountability Act (HIPAA) Privacy Rule and federal guidance on Public Welfare and the Protection of Human Subjects, and Institutional Review Board (IRB) status is exempt [36].

### Source and study populations

For commercial patients, the source population included all patients aged ≥18 years who, from July 1, 2010 through September 30, 2015, received myelosuppressive chemotherapy for solid tumors or NHL. For Medicare patients, the source population included all patients aged ≥65 years who, from July 1, 2007 through March 31, 2015, received myelosuppressive chemotherapy for solid tumors or NHL. In both the commercial and Medicare source populations, patients with < 6 months of continuous health benefits prior to initiation of chemotherapy, evidence of multiple primary cancers, or who did not meet other inclusion/exclusion criteria (as described in Additional file 1) were excluded.

From the source populations, all patients with non-metastatic breast cancer or NHL who received selected chemotherapy regimens with an intermediate/high-risk for FN were included in the study population. Selected intermediate/high-risk regimens included those commonly used in US clinical practice: docetaxel + doxorubicin + cyclophosphamide (TAC), docetaxel + cyclophosphamide (TC), and docetaxel + carboplatin + trastuzumab (TCH) for non-metastatic breast cancer; and cyclophosphamide + doxorubicin + vincristine + prednisone ± rituximab (CHOP± R) for NHL.

For each patient in the study population, each cycle of chemotherapy within the first qualifying course was characterized and use of pegfilgrastim prophylaxis on days 1–3 from the last administration of chemotherapy was ascertained in each cycle. Chemotherapy courses were limited to the first 8 cycles and were truncated if there was an unplanned switch in regimen (i.e., an unplanned change in agents administered in subsequent cycles versus the first cycle).

All patient-cycles meeting the following additional criteria were pooled for analyses: no prophylaxis with other CSF agents (i.e., use of filgrastim, tbo-filgrastim, sargramostim on the same day as chemotherapy or days 1–5 following last receipt of chemotherapy); no prophylaxis with antimicrobials; no receipt of pegfilgrastim on the same day as chemotherapy or days 4–5 following last receipt of chemotherapy; and no evidence of FN prior to administration of pegfilgrastim in that cycle.

### Febrile neutropenia

FN episodes were ascertained in each chemotherapy cycle, from the fourth day following the last receipt of chemotherapy through the last day of the cycle, using a “broad” algorithm and a “narrow” algorithm [15–17, 19, 21, 23, 24, 28, 35, 37]. For the broad algorithm, FN was ascertained in the inpatient setting based on a diagnosis (principal or secondary) of neutropenia, fever, or infection, and in the outpatient setting based on a diagnosis of neutropenia, fever, or infection and—on the same date—IV administration of antimicrobial therapy. For the narrow algorithm, FN was ascertained in the inpatient setting based on a diagnosis (principal or secondary) of neutropenia, and in the outpatient setting based on a diagnosis of neutropenia and—on the same date—administration of IV antimicrobial therapy.

### Statistical analyses

Unadjusted incidence proportions for use of pegfilgrastim prophylaxis (overall and by FN occurrence [broad algorithm] in a prior cycle) and incidence proportions for FN based on the broad algorithm (overall and for subgroups defined on receipt of pegfilgrastim prophylaxis in that cycle) were summarized on a cycle-specific basis. Corresponding unadjusted odds ratios (ORs) were estimated using generalized estimating equations (GEEs), as described below.

ORs for FN in a given cycle were also estimated for patients who did versus did not receive pegfilgrastim in that cycle, with adjustment for FN in a previous cycle, chemotherapy regimen, age, and other covariates (as described in Additional file 1) using GEEs with a binomial distribution, logistic link function, and exchangeable correlation structure. The GEE method accounts for correlation among repeated measures for the same patient (in this instance, among cycles), while controlling for both variables that are invariant as well as those that may vary across observations. These analyses were conducted for all patients in the study population using the broad and narrow algorithms for FN in cycle 1, cycle 2, cycles ≥3, the last cycle, and all cycles. All analyses were conducted at the level of the chemotherapy cycle, and analyses were conducted using commercial claims and Medicare claims separately. Other covariates were selected for inclusion in regression models via a backward selection method (*p* < 0.10), and included patient, cancer, and treatment characteristics listed in Additional file 1.

## Results

### Patient characteristics

A total of 50,778 commercial patients with non-metastatic breast cancer or NHL received 190,622 cycles of intermediate/high-risk chemotherapy during the study period and met all other criteria for inclusion. Among Medicare patients with non-metastatic breast cancer or NHL, 71,037 received 271,944 cycles of intermediate/high-risk chemotherapy during the study period and met all other criteria for inclusion. Patient characteristics were generally comparable between those who received pegfilgrastim in cycle 1 and those who did not; although some characteristics were statistically different between subgroups, the observed variation in values was not clinically meaningful (Table 1).

**Table 1:** Characteristics of study population, by use of pegfilgrasim prophylaxis in cycle 1

	Commercial		Medicare	
	No Pegfilgrastim Use	Pegfilgrastim Use	No Pegfilgrastim Use	Pegfilgrastim Use
	(*n* = 16,512)	(*n* = 34,266)	(*n* = 20,195)	(*n* = 50,842)
Patient				
Age (years)				
Mean (SD)	54.3 (10.2)	56.2 (10.9)	72.3 (5.4)	73.6 (5.9)
Female, N (%)	14,623 (89%)	29,995 (88%)	16,604 (82%)	37,811 (74%)
Chronic Comorbidities, N (%)				
Cardiovascular Disease	1403 (8%)	3851 (11%)	6124 (30%)	16,979 (33%)
Diabetes	1750 (11%)	4211 (12%)	5217 (26%)	13,022 (26%)
Liver Disease	621 (4%)	1386 (4%)	731 (4%)	1999 (4%)
Lung Disease	1290 (8%)	3157 (9%)	3033 (15%)	8053 (16%)
Renal Disease	311 (2%)	855 (2%)	1523 (8%)	4598 (9%)
Osteoarthritis	1382 (8%)	3361 (10%)	4534 (22%)	11,620 (23%)
Rheumatoid Disease	236 (1%)	561 (2%)	670 (3%)	1798 (4%)
Thyroid Disorder	2105 (13%)	4569 (13%)	4470 (22%)	11,723 (23%)
Body Weight and Nutritional Status, N (%)				
Obese	1244 (8%)	2688 (8%)	1715 (8%)	3959 (8%)
Malnutrition	71 (0%)	289 (1%)	414 (2%)	1517 (3%)
Proxies for Health Status, N (%)				
Hospice Care	38 (0%)	120 (0%)	572 (3%)	2037 (4%)
SNF	100 (1%)	244 (1%)	926 (5%)	2194 (4%)
Hospice or SNF	136 (1%)	352 (1%)	1400 (7%)	3962 (8%)
Proxies for Physical Function, N (%)				
Use of Hospital Bed	26 (0%)	78 (0%)	162 (1%)	476 (1%)
Use of Supplemental Oxygen	259 (2%)	681 (2%)	810 (4%)	2285 (4%)
Use of Walking Aid	192 (1%)	506 (1%)	772 (4%)	2455 (5%)
Use of Wheel Chair	53 (0%)	136 (0%)	378 (2%)	997 (2%)
Any of Above	487 (3%)	1256 (4%)	1807 (9%)	5268 (10%)
Use of Immunosuppressive Drugs, N (%)	5273 (32%)	11,113 (32%)	6612 (33%)	17,145 (34%)
Other Conditions/Events Prior to Chemotherapy, N (%)				
Anemia	1896 (11%)	4721 (14%)	6025 (30%)	17,970 (35%)
Neutropenia	290 (2%)	1295 (4%)	948 (5%)	4738 (9%)
Infection	7902 (48%)	17,204 (50%)	11,545 (57%)	29,868 (59%)
Recent Surgery (prior 90 days)	13,881 (84%)	28,100 (82%)	13,024 (64%)	30,427 (60%)
History of Hospitalization for Any Reason	4549 (28%)	9985 (29%)	7739 (38%)	20,374 (40%)
History of Chemotherapy	866 (5%)	2127 (6%)	1623 (8%)	4755 (9%)
History of Radiation Therapy	910 (6%)	1955 (6%)	883 (4%)	2140 (4%)
Cancer and Chemotherapy, N (%)				
Breast Cancer	13,261 (80%)	26,622 (78%)	13,305 (66%)	25,675 (50%)
TAC	389 (2%)	4179 (12%)	235 (1%)	1857 (4%)
TC	8793 (53%)	14,918 (44%)	8032 (40%)	19,493 (38%)
TCH	4079 (25%)	7525 (22%)	5038 (25%)	4325 (9%)
NHL — CHOP±R	3251 (20%)	7644 (22%)	6890 (34%)	25,167 (50%)
Year of Chemotherapy (Commercial), N (%)				
2010–2011	7150 (43%)	13,308 (39%)	–	–
2012–2013	6108 (37%)	13,108 (38%)	–	–
2014–2015	3254 (20%)	7850 (23%)	–	–
Year of Chemotherapy (Medicare), N (%)				
2007–2009	–	–	6268 (31%)	12,984 (26%)
2010–2012	–	–	8615 (43%)	21,004 (41%)
2013–2015	–	–	5312 (26%)	16,854 (33%)

*TAC* docetaxel + doxorubicin + cyclophosphamide, *TC* docetaxel + cyclophosphamide, *TCH* docetaxel + cyclophosphamide + trastuzumab, *CHOP* cyclophosphamide + doxorubicin + vincristine + prednisone with rituximab (R)

### Patterns of Pegfilgrastim prophylaxis and crude FN risk

In cycle 1, 67% of commercial patients and 72% of Medicare patients received pegfilgrastim prophylaxis (Table 2). Among commercial patients, use of pegfilgrastim prophylaxis was comparable in cycle 2 (72%), subsequent cycles (75%), and the last cycle (71%). Among Medicare patients, use of pegfilgrastim prophylaxis in cycle 2 (74%) and subsequent cycles (70%) was comparable. In the last chemotherapy cycle, however, overall pegfilgrastim use was lower (61%). Across all cycles, use of pegfilgrastim prophylaxis was somewhat higher among patients who had FN in a prior cycle (85% for commercial patients and 77% for Medicare patients).

**Table 2:** Use of pegfilgrastim prophylaxis in cycle 1, cycle 2, cycles 3+, last cycle, and all cycles, respectively, overall and by FN occurrence in a prior cycle*

	Commercial				Medicare			
		Pegfilgrastim Use				Pegfilgrastim Use		
	No. (%) in Each Category	No	Yes	Unadjusted OR (95% CI)	No. (%) in Each Category	No	Yes	Unadjusted OR (95% CI)
Cycle 1								
FN Events Prior to Cycle 1								
No	50,778 (100%)	16,512 (33%)	34,266 (67%)	–	71,037 (100%)	20,195 (28%)	50,842 (72%)	–
Yes	–	–	–	–	–	–	–	–
Cycle 2	41,769	11,719 (28%)	30,050 (72%)		59,412	15,193 (26%)	44,219 (74%)	
FN Events Prior to Cycle 2								
No	40,123 (96%)	11,482 (29%)	28,641 (71%)	–	55,488 (93%)	14,483 (26%)	41,005 (74%)	–
Yes	1646 (4%)	237 (14%)	1409 (86%)	2.4 (2.1–2.7)	3924 (7%)	710 (18%)	3214 (82%)	1.6 (1.5–1.7)
Cycles ≥3	98,075	24,834 (25%)	73,241 (75%)		141,495	43,012 (30%)	98,483 (70%)	
FN Events Prior to Cycle of Interest								
No	91,744 (94%)	23,875 (26%)	67,869 (74%)	–	126,424 (89%)	39,360 (31%)	87,064 (69%)	–
Yes	6331 (6%)	959 (15%)	5372 (85%)	2.0 (1.8–2.1)	15,071 (11%)	3652 (24%)	11,419 (76%)	1.4 (1.4–1.5)
Last Cycle	34,860	10,188 (29%)	24,672 (71%)		51,989	20,099 (39%)	31,890 (61%)	
FN Events Prior to Last Cycle								
No	32,541 (93%)	9774 (30%)	22,767 (70%)	–	46,115 (89%)	18,215 (39%)	27,900 (61%)	–
Yes	2319 (7%)	414 (18%)	1905 (82%)	2.0 (1.8–2.2)	5874 (11%)	1884 (32%)	3990 (68%)	1.4 (1.3–1.5)
All Cycles	190,622	53,065 (28%)	137,557 (72%)		271,944	78,400 (29%)	193,544 (71%)	
FN Events Prior to Cycle of Interest								
No	182,645 (96%)	51,869 (28%)	130,776 (72%)	–	252,949 (93%)	74,038 (29%)	178,911 (71%)	–
Yes	7977 (4%)	1196 (15%)	6781 (85%)	2.2 (2.1–2.4)	18,995 (7%)	4362 (23%)	14,633 (77%)	1.4 (1.3–1.4)

*FN* febrile neutropenia

*Only consecutive qualifying cycles, beginning with cycle 1, were considered in this analysis (e.g., in identifying FN events [broad definition] in a prior cycle of the course of interest)

The unadjusted FN incidence among commercial and Medicare patients was 3.0 and 6.2% across all cycles, 5.2 and 9.6% in cycle 1, 2.3 and 5.2% in cycle 2, and 2.1 and 5.0% in subsequent cycles, respectively (Table 3). For both commercial and Medicare patients, FN incidence was generally lower among patients who received pegfilgrastim prophylaxis versus those who did not.

**Table 3:** Crude incidence proportions for FN (broad definition) in cycle 1, cycle 2, cycles 3+, last cycle, and all cycles, respectively, by receipt of pegfilgrastim prophylaxis*

	Commercial				Medicare			
		FN in Cycle				FN in Cycle		
	No. (%) in Each Category	No	Yes	Unadjusted OR (95% CI)	No. (%) in Each Category	No	Yes	Unadjusted OR (95% CI)
Cycle 1	50,778	48,157 (95%)	2621 (5%)		71,037	64,249 (90%)	6788 (10%)	
Use of Pegfilgrastim Prophylaxis								
No	16,512 (33%)	15,234 (92%)	1278 (8%)	–	20,195 (28%)	17,901 (89%)	2294 (11%)	–
Yes	34,266 (67%)	32,923 (96%)	1343 (4%)	2.1 (1.9–2.2)	50,842 (72%)	46,348 (91%)	4494 (9%)	1.3 (1.3–1.4)
Cycle 2	41,769	40,810 (98%)	959 (2%)		59,412	56,343 (95%)	3069 (5%)	
Use of Pegfilgrastim Prophylaxis								
No	11,719 (28%)	11,353 (97%)	366 (3%)	–	15,193 (26%)	14,282 (94%)	911 (6%)	–
Yes	30,050 (72%)	29,457 (98%)	593 (2%)	1.6 (1.4–1.8)	44,219 (74%)	42,061 (95%)	2158 (5%)	1.2 (1.1–1.3)
Cycles ≥3	98,075	96,006 (98%)	2069 (2%)		141,495	134,363 (95%)	7132 (5%)	
Use of Pegfilgrastim Prophylaxis								
No	24,834 (25%)	24,229 (98%)	605 (2%)	–	43,012 (30%)	40,842 (95%)	2170 (5%)	–
Yes	73,241 (75%)	71,777 (98%)	1464 (2%)	1.2 (1.1–1.3)	98,483 (70%)	93,521 (95%)	4962 (5%)	1.0 (1.0–1.1)
Last Cycle	34,860	33,426 (96%)	1434 (4%)		51,989	45,392 (87%)	6597 (13%)	
Use of Pegfilgrastim Prophylaxis								
No	10,188 (29%)	9705 (95%)	483 (5%)	–	20,099 (39%)	17,851 (89%)	2248 (11%)	–
Yes	24,672 (71%)	23,721 (96%)	951 (4%)	1.2 (1.1–1.4)	31,890 (61%)	27,541 (86%)	4349 (14%)	0.8 (0.8–0.8)
All Cycles	190,622	184,973 (97%)	5649 (3%)		271,944	254,955 (94%)	16,989 (6%)	
Use of Pegfilgrastim Prophylaxis								
No	53,065 (28%)	50,816 (96%)	2249 (4%)	–	78,400 (29%)	73,025 (93%)	5375 (7%)	–
Yes	137,557 (72%)	134,157 (98%)	3400 (2%)	1.7 (1.7–1.8)	193,544 (71%)	181,930 (94%)	11,614 (6%)	1.2 (1.1–1.2)

*FN* febrile neutropenia

*Only consecutive qualifying cycles, beginning with cycle 1, were considered in this analysis

### Multivariable analysis of FN

After adjustment for differences in age, chemotherapy regimen, and other covariates, odds of FN over all cycles (broad algorithm) were 2.1 times higher (95% CI 2.0–2.3) among commercial patients and 1.5 times higher (95% CI 1.4–1.5) among Medicare patients who did not receive pegfilgrastim prophylaxis versus those who did (Table 4). In cycle 1, FN odds were 2.6 times higher (95% CI 2.3–2.8) among commercial patients and 1.6 times higher (95% CI 1.5–1.7) among Medicare patients. After adjustment for the aforementioned factors as well as FN in a prior cycle, FN odds in cycle 2 and subsequent cycles were similarly elevated for those not receiving prophylaxis among both commercial and Medicare patients.

**Table 4:** Adjusted odds ratios for FN in cycle 1, cycle 2, cycles 3+, last cycle, and all cycles, respectively (Broad Definition of FN)

	Adjusted Odds Ratio for FN (95% CI, *p*-value)*				
	Cycle 1	Cycle 2	Cycles ≥3	Last Cycle	All Cycles
Commercial					
Independent Variables					
No PEG Prophylaxis	2.6 (2.3–2.8, < 0.001)	1.9 (1.6–2.2, < 0.001)	1.5 (1.4–1.7, < 0.001)	1.5 (1.4–1.7, < 0.001)	2.1 (2.0–2.3, < 0.001)
FN in Prior Cycle	–	3.2 (2.6–3.9, < 0.001)	3.5 (3.2–4.0, < 0.001)	2.4 (2.0–2.8, < 0.001)	2.4 (2.2–2.6, < 0.001)
Regimen					
TAC	2.9 (2.6–3.3, < 0.001)	2.6 (1.7–4.1, < 0.001)	1.7 (1.4–1.9, < 0.001)	3.3 (2.4–4.8, < 0.001)	2.7 (2.2–3.2, < 0.001)
TC	–	–	–	–	–
TCH	1.1 (1.0–1.2, 0.229)	2.2 (1.4–3.3, < 0.001)	1.1 (1.0–1.2, 0.266)	2.1 (1.5–2.9, < 0.001)	1.4 (1.2–1.6, < 0.001)
CHOP±R	1.3 (1.2–1.5, < 0.001)	4.1 (1.9–9.0, < 0.001)	1.7 (1.5–1.9, < 0.001)	4.3 (2.4–7.7, < 0.001)	2.6 (1.9–3.5, < 0.001)
Age					
18–49	–	–	–	–	–
50–64	1.0 (0.9–1.1, 0.377)	1.0 (0.9–1.2, 0.884)	1.1 (1.0–1.2, 0.242)	1.3 (1.1–1.4, 0.002)	1.0 (1.0–1.1, 0.409)
65–74	1.3 (1.2–1.5, < 0.001)	1.0 (0.8–1.2, 0.881)	1.5 (1.3–1.8, < 0.001)	1.7 (1.4–2.1, < 0.001)	1.3 (1.2–1.4, < 0.001)
≥ 75	1.7 (1.4–2.1, < 0.001)	1.2 (0.9–1.7, 0.200)	2.1 (1.8–2.6, < 0.001)	3.4 (2.7–4.3, < 0.001)	1.7 (1.5–2.0, < 0.001)
Medicare					
Independent Variables					
No PEG Prophylaxis	1.6 (1.5–1.7, < 0.001)	1.6 (1.5–1.8, < 0.001)	1.3 (1.3–1.4, < 0.001)	1.0 (0.9–1.0, 0.507)	1.5 (1.4–1.5, < 0.001)
FN in Prior Cycle	–	3.2 (2.9–3.5, < 0.001)	2.9 (2.7–3.1, < 0.001)	1.7 (1.6–1.8, < 0.001)	2.1 (2.1–2.2, < 0.001)
Regimen					
TAC	2.5 (2.2–2.9, < 0.001)	1.9 (1.5–2.4, < 0.001)	2.0 (1.7–2.3, < 0.001)	2.2 (1.9–2.6, < 0.001)	2.1 (1.9–2.3, < 0.001)
TC	–	–	–	–	–
TCH	0.8 (0.7–0.9, < 0.001)	0.9 (0.8–1.0, 0.127)	0.8 (0.7–0.9, < 0.001)	1.0 (0.9–1.2, 0.387)	0.8 (0.7–0.8, < 0.001)
CHOP±R	1.5 (1.4–1.5, < 0.001)	1.8 (1.7–2.0, < 0.001)	1.9 (1.8–2.0, < 0.001)	1.8 (1.7–2.0, < 0.001)	1.6 (1.6–1.7, < 0.001)
Age					
65–74	–	–	–	–	–
75–84	1.2 (1.1–1.3, < 0.001)	1.0 (0.9–1.1, 0.709)	1.1 (1.1–1.2, < 0.001)	1.2 (1.1–1.3, < 0.001)	1.1 (1.1–1.2, < 0.001)
≥ 85	1.4 (1.3–1.5, < 0.001)	1.2 (1.1–1.4, < 0.001)	1.4 (1.3–1.5, < 0.001)	1.8 (1.6–1.9, < 0.001)	1.4 (1.3–1.4, < 0.001)

*FN* febrile neutropenia, *PEG* pegfilgrastim, *TC* docetaxel + cyclophosphamide, *TAC* docetaxel + doxorubicin + cyclophosphamide, *TCH* docetaxel + cyclophosphamide + trastuzumab, *CHOP* cyclophosphamide + doxorubicin + vincristine + prednisone, *R*, rituximab

*Adjusted for other characteristics of patients listed in Table 1; additional covariates selected via backward selection method

For commercial patients, odds of FN across all cycles were substantially higher among those who had an FN event in a prior cycle (OR = 2.4; 95% CI 2.2–2.6), were generally highest with TAC (OR [vs TC] = 2.7; 95% CI 2.2–3.2) and CHOP±R (OR [vs TC] = 2.6; 95% CI 1.9–3.5), and increased with age (65–74 [vs 18–49]: OR = 1.3; 95% CI 1.2–1.4; ≥75 [vs 18–49]: OR = 1.7, 95% CI 1.5–2.0). Similar patterns were seen among Medicare patients: odds of FN across all cycles were substantially higher among those who had an FN event in a prior cycle (OR = 2.1 [95% CI 2.1–2.2]), were highest with TAC (OR [vs TC] = 2.1 [95% CI 1.9–2.3]) and CHOP±R (OR vs TC = 1.6; 95% CI 1.6–1.7), and increased with age (75–84 [vs 65–74]: OR = 1.1 [95% CI 1.1–1.2]; ≥85 [vs 65–74]: OR = 1.4 [95% CI 1.3–1.4]). For both commercial and Medicare patients, results based on the narrow algorithm for FN suggest that elevated odds of FN among patients not receiving prophylaxis were even higher (Table 5).

**Table 5:** Adjusted odds ratios for FN in cycle 1, cycle 2, cycles 3+, last cycle, and all cycles, respectively (Narrow Definition of FN)

	Adjusted Odds Ratio for FN (95% CI, p-value)*				
	Cycle 1	Cycle 2	Cycles ≥3	Last Cycle	All Cycles
Commercial					
Independent Variables					
No PEG Prophylaxis	4.2 (3.8–4.7, < 0.001)	4.1 (3.3–4.9, < 0.001)	3.3 (2.9–3.8, < 0.001)	3.2 (2.7–3.8, < 0.001)	4.2 (3.9–4.5, < 0.001)
FN in Prior Cycle	–	5.1 (3.7–7.0, < 0.001)	5.9 (4.9–7.0, < 0.001)	3.9 (3.0–5.0, < 0.001)	3.1 (2.6–3.5, < 0.001)
Regimen					
TAC	4.4 (3.8–5.1, < 0.001)	2.2 (1.6–3.2, < 0.001)	3.0 (2.4–3.8, < 0.001)	2.7 (2.0–3.6, < 0.001)	4.1 (3.3–5.2, < 0.001)
TC	–	–	–	–	–
TCH	1.0 (0.9–1.1, 0.963)	1.4 (1.1–1.8, 0.009)	1.2 (1.0–1.4, 0.130)	1.2 (0.9–1.5, 0.191)	1.3 (1.0–1.6, 0.040)
CHOP±R	1.4 (1.2–1.6, < 0.001)	2.0 (1.5–2.7, < 0.001)	3.5 (2.9–4.4, < 0.001)	1.7 (1.4–2.1, < 0.001)	3.0 (2.0–4.5, < 0.001)
Age					
18–49	–	–	–	–	–
50–64	1.1 (1.0–1.2, 0.216)	1.1 (0.9–1.4, 0.332)	1.0 (0.9–1.2, 0.671)	1.3 (1.1–1.6, 0.010)	1.1 (1.0–1.2, 0.207)
65–74	1.5 (1.3–1.8, < 0.001)	1.2 (0.8–1.6, 0.354)	1.4 (1.1–1.8, 0.002)	1.6 (1.2–2.1, 0.003)	1.4 (1.3–1.6, < 0.001)
≥ 75	2.1 (1.7–2.7, < 0.001)	1.7 (1.1–2.6, 0.012)	2.0 (1.5–2.6, < 0.001)	4.0 (2.9–5.5, < 0.001)	2.1 (1.7–2.4, < 0.001)
Medicare					
Independent Variables					
No PEG Prophylaxis	2.0 (1.9–2.2, < 0.001)	2.2 (2.0–2.5, < 0.001)	1.6 (1.5–1.8, < 0.001)	1.3 (1.2–1.4, < 0.001)	1.9 (1.8–2.0, < 0.001)
FN in Prior Cycle	–	4.6 (4.0–5.3, < 0.001)	4.1 (3.8–4.4, < 0.001)	2.4 (2.1–2.6, < 0.001)	2.7 (2.5–2.9, < 0.001)
Regimen					
TAC	3.1 (2.7–3.7, < 0.001)	2.5 (1.9–3.5, < 0.001)	2.8 (2.3–3.5, < 0.001)	2.5 (2.0–3.1, < 0.001)	2.7 (2.4–3.0, < 0.001)
TC	–	–	–	–	–
TCH	0.6 (0.6–0.7, < 0.001)	0.7 (0.5–0.9, 0.001)	0.6 (0.5–0.8, < 0.001)	0.7 (0.6–0.8, < 0.001)	0.6 (0.5–0.6, < 0.001)
CHOP±R	1.6 (1.5–1.7, < 0.001)	2.1 (1.9–2.5, < 0.001)	2.9 (2.6–3.2, < 0.001)	2.1 (1.9–2.3, < 0.001)	1.9 (1.8–2.0, < 0.001)
Age					
65–74	–	–	–	–	–
75–84	1.2 (1.1–1.3, < 0.001)	1.0 (0.8–1.1, 0.759)	1.1 (1.0–1.2, 0.011)	1.2 (1.1–1.4, < 0.001)	1.1 (1.1–1.2, < 0.001)
≥ 85	1.4 (1.3–1.6, < 0.001)	1.1 (1.0–1.3, 0.047)	1.2 (1.1–1.4, < 0.001)	1.6 (1.5–1.8, < 0.001)	1.3 (1.3–1.4, < 0.001)

*FN* febrile neutropenia, *PEG* pegfilgrastim, *TC* docetaxel + cyclophosphamide, *TAC* docetaxel + doxorubicin + cyclophosphamide, *TCH* docetaxel + cyclophosphamide + trastuzumab, *CHOP* cyclophosphamide + doxorubicin + vincristine + prednisone, R rituximab

*Adjusted for other characteristics of patients listed in Table 1; additional covariates selected via backward selection method

## Discussion

In this retrospective observational cohort study of patients with non-metastatic breast cancer or NHL who received chemotherapy with an intermediate to high risk for FN, we examined patterns of pegfilgrastim prophylaxis across cycles of chemotherapy, the influence of FN in one chemotherapy cycle on prophylaxis in subsequent cycles, and the incidence of FN among patients who did and did not receive prophylaxis. The findings from this examination—which were based on data from 50,778 commercial patients who received 190,622 cycles of chemotherapy and 71,037 Medicare patients who received 271,944 cycles— suggest that not only does a large minority of patients for whom prophylaxis is recommended fail to receive it (beginning in the first cycle), but that those patients have significantly higher odds of FN than patients who receive pegfilgrastim prophylaxis.

We found that approximately one in every three patients in this study did not receive pegfilgrastim prophylaxis in cycle 1—when FN risk is highest—and comparable proportions did not receive prophylaxis in subsequent cycles, thus exposing patients to a potentially fatal yet preventable complication of myelosuppressive chemotherapy [38]. We also found that while a history of FN does appear to increase the odds of receiving prophylactic coverage, more than one in seven commercial patients and one in four Medicare patients did not receive prophylaxis in a given cycle despite having FN in a prior cycle. Finally, we found that the adjusted incidence of FN in a given cycle was significantly higher among patients in our study population who did not receive prophylaxis in that cycle, highlighting the effectiveness of pegfilgrastim. We note that our findings are based on two large samples of patients with non-metastatic breast cancer and NHL who received chemotherapy regimens that are among the most commonly used in current clinical practice, and are largely consistent with the limited evidence that is currently available [39–47].

Two systematic reviews of randomized, controlled clinical trials comparing G-CSF prophylaxis with no prophylaxis showed significantly reduced risk of FN with pegfilgrastim [39, 43]. Similar results have been observed in real-world data. In Hershman et al., which included 3123 randomly selected patients with solid tumors and lymphomas treated at 99 community practices in 2003, FN risk during the chemotherapy course was reported to be two-times higher among patients who did not receive primary prophylaxis (i.e., in cycle 1) versus those who did (adjusted OR = 2.0 [95% CI 1.4–2.9]) [46]. In a study of 239 women receiving adjuvant chemotherapy for early-stage breast cancer at a single clinic from 2009 to 2011, the FN odds ratio for patients not receiving G-CSF primary prophylaxis (versus those receiving prophylaxis) was 2.6 (*p* = 0.002) [44]. The results of two other smaller studies were similar [41, 45]. Accordingly, the results of these studies provide robust clinical and real-world evidence regarding the effectiveness of pegfilgrastim prophylaxis in the prevention of chemotherapy-induced FN among cancer patients of all ages.

Our results also are noteworthy given that clinical practice guidelines recommend administration of G-CSF in the first cycle when the risk of FN is > 20%, and in subsequent cycles after FN or a dose-limiting neutropenic event where no prior G-CSF has been used. Unfortunately, the reasons why prophylaxis was not administered to so many patients in this study are unknown. While it is possible that other steps were taken to reduce the risk of FN, such as chemotherapy dose reductions (which are unobservable in the study database), it is also possible that patients were reluctant to return to the clinic to receive a pegfilgrastim injection on the day after chemotherapy [48, 49]. Regardless of the reasons or the steps other than prophylaxis that were taken to prevent FN, our results demonstrate that patients with a history of FN were substantially more likely to experience a subsequent FN episode during their chemotherapy course, consistent with previous studies [38, 50].

There are several notable limitations to our study. A diagnosis code for FN does not exist, and thus FN was ascertained using operational algorithms and codes for neutropenia, fever, and infection that appeared during the pre-defined exposure period. We note that the recording of these diagnosis codes—especially those appearing earlier in the cycle (e.g., day 14 or earlier)—during the chemotherapy course increases the probability that the condition (i.e., neutropenia, infection, and/or fever) is associated with chemotherapy. In addition, ascertainment of FN in hospital was based on diagnosis codes alone as data on inpatient drug utilization are not available in the study databases. Because results from lab and other tests are unavailable in the study databases, and because other information (e.g., chemotherapy dose) is unavailable, not all FN risk factors were considered in analyses described herein. Because the validity of algorithms for identifying primary tumor type, metastatic disease, and comorbidity profiles has not been formally evaluated, their accuracy is unknown. Thus, to the extent that there may be unobserved systematic differences between patients who did (vs. did not) receive pegfilgrastim prophylaxis, study results may be confounded.

While approximately 20% of the commercial population and 40% of the Medicare population received an intermediate-risk chemotherapy regimen, it is likely that most of those patients would be classified as high-risk when considering patient risk factors (e.g., age > 65 years, comorbidities) and thus recommended to receive G-CSF prophylaxis [51]. It is possible, however, that a small percentage of patients in the study population may have had a projected risk of FN < 20%. While over 95% of CSF prophylaxis patients received pegfilgrastim, a small percentage received one of the daily agents (principally filgrastim) and thus overall CSF use is somewhat higher than reported estimates. While the study period overlapped for a few months with the availability of the Neulasta® Onpro® kit—an on-body injector that is applied 1 day and delivers pegfilgrastim approximately 27 h later—these patients were excluded from analyses if their pegfilgrastim administration was identified as having occurred on the same day as chemotherapy. Based on the timing of the data relative to Onpro® availability, the net impact of such exclusions was likely small. Because our study population was limited to patients with non-metastatic breast cancer or NHL who received selected intermediate/high-risk chemotherapy regimens, study results may not be generalizable to other cancers or other regimens.

## Conclusions

In this retrospective evaluation of non-metastatic breast cancer and NHL patients receiving select chemotherapy regimens with an intermediate/high-risk for FN, a sizeable portion of patients did not receive G-CSF prophylaxis, and an important minority did not receive G-CSF prophylaxis in cycles following FN. Patients not receiving G-CSF prophylaxis had a markedly higher incidence of FN versus those who received G-CSF prophylaxis. Accordingly, appropriate use of G-CSF prophylaxis may reduce exposure to a potentially fatal but largely preventable complication of myelosuppressive chemotherapy.

## Additional file

Additional file 1: Online supplement:study methods. (DOCX 143 kb)

## Data Availability

The data are proprietary, provided by third-party vendors, and the authors do not have permission to disseminate these data without approval of the vendors.

## Abbreviations

ANC: Absolute neutrophil count
CHOP±R: Cyclophosphamide + doxorubicin + vincristine + prednisone ± rituximab
CMS: Centers for Medicare and Medicaid Services
CSF: Colony-stimulating factor
FN: Febrile neutropenia
G-CSF: Granulocyte colony-stimulating factor
GEE: Generalized estimating equations
HIPAA: Health Insurance Portability and Accountability Act
IRB: Institutional Review Board
NHL: Non-Hodgkin’s lymphoma
OR: Odds ratio
PAI: Policy Analysis Inc.
RIFs: Research Identifiable Files
TAC: Docetaxel + doxorubicin + cyclophosphamide
TC: Docetaxel + cyclophosphamide
TCH: Docetaxel + carboplatin + trastuzumab
