# Supplementary material for: Use and effectiveness of pegfilgrastim prophylaxis in US clinical practice:a retrospective observational study
Source: BMC Cancer. 2019 Aug 9;19:792. doi: 10.1186/s12885-019-6010-9 (PMC6688232; doi:10.1186/s12885-019-6010-9)
Supplement: Supplementary file 1 — Online supplement:study methods. (DOCX 143 kb) [file 12885_2019_6010_MOESM1_ESM.docx]

ONLINE SUPPLEMENT:

**STUDY METHODS**

# METHODS

## Study Design

This study employed a retrospective cohort design and data from two large integrated US private healthcare claims repositories (“commercial”) as well as the Medicare Claims Research Identifiable Files (“Medicare”). Patient-level claims information was employed in analyses, which were conducted using data from the pooled commercial repositories and Medicare claims files, respectively. The study databases were deidentified prior to their release to the study investigators, and their use for health services research was fully compliant with the Health Insurance Portability and Accountability Act (HIPAA) Privacy Rule and federal guidance on Public Welfare and the Protection of Human Subjects (45 CFR 46 §46.101). Accordingly, this research is exempt from IRB review.

A schematic of the study design is set forth in Appendix A. Operational algorithms and corresponding codes (diagnosis, procedure, drug) employed to identify study variables are described below and set forth in Appendices B-G; ICD-10-CM codes are available upon request.

## Data Source Population

**Commercial.** The data source population comprised (principally) persons who had private employer-sponsored healthcare coverage, along with their spouses and dependents. Elderly persons who were Medicare-eligible and had elected to enroll in the Medicare Advantage Program or in a Medicare supplemental plan—and thus received their healthcare coverage, in part or in full, through a private health plan—were also included in the data source population.

**Medicare.** The data source population comprises (principally) elderly (≥65 years of age) persons who were enrolled in the traditional Medicare fee-for-service program (i.e., Medicare Part A and Medicare Part B). Individuals who received Social Security Disability Insurance (SSDI) for ≥24 months, persons with end-stage renal disease (ESRD), and persons with amyotrophic lateral sclerosis (ALS), regardless of age, were also included in the data source population.

## Data Source(s)

**Commercial.** The two study repositories―Truven Health Analytics MarketScan® Commercial Claims and Encounters and Medicare Supplemental and Coordination of Benefits Databases (“MarketScan Database”); IMS LifeLink™ PharMetrics Plus Health Plan Claims Database (“PharMetrics Plus Database”)―comprise medical (i.e., facility and professional service) and outpatient pharmacy claims from a large number of participating health plans. Data extracts from the two repositories spanned the period from January 1, 2010, through March 31, 2016.

The MarketScan Database includes information (primarily) from employer-sponsored plans throughout the US that provide health benefits to >15 million persons annually, including employees, their spouses, and their dependents, 10% of whom are aged ≥65 years. The PharMetrics Plus Database includes information from >75 US private health plans providing healthcare coverage to a geographically diverse population of >15 million persons annually; 4% of plan members are ≥65 years of age. The MarketScan and PharMetrics Plus Databases include information from unique health plans/organizations.

Data available from each facility and professional-service claim include dates and places of service, diagnoses, procedures performed/services rendered, and quantity of services (professional-service claims only). Data available for each outpatient pharmacy claim include the drug (class) dispensed, dispensing date, quantity dispensed, and number of days supplied. Medical and pharmacy claims also include amounts paid (i.e., reimbursed) by health plans, as well as by patients, for healthcare services rendered. Selected demographic and eligibility information (including age, sex, geographic region of residence, and dates of plan eligibility) is available for all health plan enrollees in the databases. All data can be arrayed to provide a detailed chronology of medical and pharmacy services used by each plan member over time.

**Medicare.** Medicare Claims Research Identifiable Files (RIFs) comprise inpatient (i.e., hospital inpatient provider), outpatient (i.e., institutional outpatient provider), physicians/suppliers (i.e., non-institutional provider [Carrier]), home health agencies (HHA), skilled nursing facilities (SNF), hospice, durable medical equipment (DME), and the Part D (i.e., prescription drug) program final-action claims for services provided to beneficiaries enrolled in the traditional fee-for-service program. A Master Beneficiary Summary File containing demographic and enrollment information for all Medicare beneficiaries enrolled in the fee-for-service program was employed to characterize the demographic profile (e.g., age, sex) and periods of eligibility for medical/drug benefits. The data extract spanned the period from January 1, 2007 through September 30, 2015.

Data available from each inpatient, outpatient, physician/supplier, HHA, SNF, hospice, and DME claim include dates and places of service, diagnoses, procedures performed/services rendered, and quantity of services (professional-service claims only). Data available for each Part D claim include the drug (class) dispensed, dispensing date, quantity dispensed, and number of days supplied. Inpatient, outpatient, physicians/suppliers, HHA, SNF, hospice, DME, and Part D claims also include amounts paid (i.e., reimbursed) by Medicare, as well as by patients, for healthcare services rendered. Selected demographic and eligibility information (including age, sex, beneficiary zip code, dates of Medicare eligibility) is available for all Medicare FFS enrollees in the data extract. All data can be arrayed to provide a detailed chronology of medical and pharmacy services used by each beneficiary over time.

## Patient Eligibility

For commercial patients, the source population comprised all patients aged ≥18 years who, between July 1, 2010 and September 30, 2015, initiated ≥1 course of myelosuppressive chemotherapy for a single primary solid tumor or NHL, and met minimum health benefit eligibility criteria. For Medicare patients, the source population comprised all patients aged ≥65 years who, between July 1, 2007 and March 31, 2015, initiated ≥1 course of myelosuppressive chemotherapy for a single primary solid tumor or NHL, and met minimum health benefit eligibility criteria.

For each patient in the source populations, the first unique observed course of chemotherapy, and each cycle within the first course, was identified. From the source populations, all patients who received chemotherapy regimens with an intermediate/high-risk for FN and that are commonly used in US clinical practice were selected for inclusion in the study populations.^1-4^ Patient-cycles that met all inclusion/exclusion criteria set forth below were pooled for analyses.

### Inclusion Criteria

Patients who satisfied the following criteria were selected for inclusion in the source population:

- Commercial:
  - Receipt of ≥1 course of myelosuppressive chemotherapy for a solid tumor or NHL between July 1, 2010 and September 30, 2015;
  - Evidence of a primary solid tumor or NHL; and
  - Aged ≥18 years at the time of chemotherapy initiation.
- Medicare:
  - Receipt of ≥1 course of myelosuppressive chemotherapy for a solid tumor or NHL between July 1, 2007 and March 31, 2015;
  - Evidence of a primary solid tumor or NHL; and
  - Aged ≥65 years at the time of chemotherapy initiation.

From the source populations, all patients who received selected intermediate/high-risk chemotherapy regimens (excluding those that were administered on a weekly basis, based on the interval between the first and second cycles) were flagged for inclusion in the study population. The list of cancer/regimen combinations of interest included:

- Non-metastatic breast cancer:
  - TAC: docetaxel + doxorubicin + cyclophosphamide
  - TC: docetaxel + cyclophosphamide
  - TCH: docetaxel + carboplatin + trastuzumab
- NHL – CHOP±R: cyclophosphamide + doxorubicin + vincristine + prednisone ± rituximab

**Primary and Secondary Cancers.** Presence of solid tumors and NHL were identified based on ≥2 encounters (≥7 days apart) with a qualifying ICD-9-CM/ICD-10-CM diagnosis code (Appendix B) during the period beginning 30 days prior to the date of chemotherapy initiation and ending 30 days thereafter. Presence of metastasis was identified on the basis of ≥1 diagnosis code (ICD-9-CM 197-198; ICD-10-CM C78-C79) on inpatient claims or ≥2 diagnosis codes on outpatient claims (excluding those for laboratory services) on different days during the 1-year period prior to chemotherapy initiation.

**Chemotherapy Courses and Cycles.** For each cancer chemotherapy patient, each unique cycle within the first observed course of chemotherapy was identified. The first chemotherapy cycle (of the first course) was defined as beginning with the date of initiation of chemotherapy and ending with the first service date for the next administration of chemotherapy administration (as evidenced by an encounter with a corresponding HCPCS or ICD-9-CM/ICD-10-CM code) occurring at least 7 days—but no more than 59 days—after the date of initiation of chemotherapy. If a second chemotherapy cycle did not commence prior to day 60, or if there was an unplanned change in the chemotherapy regimen (i.e., based on expert opinion regarding agents received in first cycle versus subsequent cycles), both the first cycle of chemotherapy and the course of chemotherapy were considered to have been completed 35 days following the beginning of the cycle or on the date of change in the regimen, as appropriate. The second and all subsequent cycles of chemotherapy in each unique chemotherapy course during the period of interest, were similarly defined. Only consecutive qualifying cycles, beginning with cycle 1 and ending with cycle 8, were considered in this analysis.

Only myelosuppressive chemotherapy agents (Appendix C) were considered in characterizing courses and cycles therein. Chemotherapy courses were characterized based on observed patterns of administration using information captured in claims—including corresponding procedure/revenue codes (HCPCS, ICD-9-CM/ICD-10-CM, Uniform Bill-92 [UB-92]) and dates of service—as well as clinical expertise regarding the regimens and their patterns of use in clinical practice.

**Chemotherapy Regimens.** Chemotherapy regimens were ascertained based on a review of all HCPCS Level II codes for parenterally administered antineoplastic agents (myelosuppressive and non-myelosuppressive) on claims with service dates within seven days of the start of each cycle of chemotherapy. Chemotherapy regimens were characterized based on the agents received during the course as well as based on cycle periodicity (i.e., every two weeks [Q2W], every three weeks [Q3W], every four weeks [Q4W], based on the observed interval between the first and second cycles), as feasible.

**Pegfilgrastim Prophylaxis.** Pegfilgrastim prophylaxis was ascertained based on a review of all HCPCS Level II codes (C9119, S0135, J2505) on medical claims with service dates on days 1-3 from the last administration of chemotherapy in each cycle.

### Exclusion Criteria

Patients were excluded from the source populations if there was/were:

- Any gaps in their eligibility for comprehensive medical and drug benefits during the 6-month (“pretreatment”) period prior to initiation of their index chemotherapy course;
- Evidence of ≥2 primary solid cancers within (i.e., +/-) 30 days of the date of chemotherapy initiation, except for patients with evidence of a primary cancer(s) and metastatic disease at the same site, as described below; or
- Evidence of hematopoietic stem cell or bone marrow transplantation prior to or during receipt of chemotherapy.

Patient-cycles were excluded from the study if there was/were:

- Evidence of other CSFs (i.e., filgrastim, tbo-filgrastim, and sargramostim) or antibiotics administered prophylactically;
- Receipt of pegfilgrastim on the same day as chemotherapy or days 4-5 following last receipt of chemotherapy;
- Evidence of pegfilgrastim prophylaxis based on outpatient pharmacy claims with a corresponding code from the NDC system; or
- An FN episode that occurred prior to administration of pegfilgrastim during the cycle.

Patients who have evidence of ≥2 primary cancers (e.g., breast cancer and lung cancer) and evidence of distant metastatic disease to ≥1 of these sites (e.g., lung) were classified as having a single primary tumor (i.e., breast cancer metastatic to the lung) and thus were retained in the source population (Appendix D). (The assumption in such cases is that metastatic disease in the lung was miscoded as a primary malignancy.)

Other CSF prophylaxis use was ascertained based on a review of all codes from the HCPCS Level II system (filgrastim [J1440; J1441; J1442], tbo-filgrastim [J1446, J1447], sargramostim [J2820]) and NDC system on medical/pharmacy claims with service dates on or up to three days from chemotherapy completion. Evidence of receipt of oral antibiotic prophylaxis was identified based on corresponding drug codes from the NDC system that are present on pharmacy claims with service dates on or up to three days from chemotherapy completion.

## Definitions

### Definitions of Time Periods

#### Study Period

**Commercial.** The study period spanned January 1, 2010 through March 31, 2016.

**Medicare.** The study period spanned January 1, 2007 to September 30, 2015.

#### Baseline Period

The baseline period for evaluating characteristics of patients, their cancer, and their treatment spanned the 12-month pre-chemotherapy period.

#### Study Follow-up Period

Follow-up for ascertainment of FN began on the fourth day from completion of chemotherapy and ended on the last day of the chemotherapy cycle.

#### Endpoint(s)/Outcomes(s) Assessment

**Febrile Neutropenia Requiring Inpatient Care.** In the “broad” definition, FN requiring inpatient care (“Inpatient FN”) was identified based on an inpatient admission with a diagnosis (principal or secondary) of neutropenia (ICD-9-CM 288.0x; ICD-10-CM D70.x), or fever (ICD-9-CM 780.6; ICD-10-CM R50.2, R50.9, R50.81-R50.84, R68.0, R68.83), or infection (Appendix E). A “narrow” definition for FN comprising inpatient encounters with a diagnosis (principal or secondary) of neutropenia was evaluated in sensitivity analyses. Hospitalizations were identified on a cycle-specific basis using acute-care facility inpatient claims with admission dates anytime on/between the fourth day following completion of chemotherapy administration and the last day of the chemotherapy cycle.

**Febrile Neutropenia Requiring Outpatient Care.** In the “broad” definition, FN requiring outpatient care only (“Outpatient FN”) was ascertained based on an encounter in the outpatient setting (e.g., physician’s office, emergency department, home) with a diagnosis of neutropenia, or fever, or infection and—on the same date—a HCPCS Level II (i.e., CPT) code for IV administration of antimicrobial therapy. A “narrow” definition for FN comprising outpatient encounters with a diagnosis (principal or secondary) of neutropenia and—on the same date—IV antimicrobial therapy was evaluated in sensitivity analyses. Such encounters that precede or follow a hospitalization during the same cycle of chemotherapy were considered as separate outpatient episodes (i.e., they were classified as part of the episode of FN requiring inpatient care). Outpatient care episodes were identified on a cycle-specific basis using outpatient claims with dates of service on/between the fourth day following completion of chemotherapy administration and the last day of the chemotherapy cycle.

#### Other Study Variables

Characteristics described below represent many of those listed by NCCN as important risk factors for FN. It was anticipated that demographic characteristics would be available for nearly all study subjects. All other characteristics were defined based on the presence of specific data (e.g., diagnosis codes, procedure codes); the absence of such data was assumed to indicate the absence of the characteristic captured by the variable. While the accuracy of variables capturing healthcare encounters and use of pharmacotherapy (e.g., G-CSF and antimicrobial agents) is expected to be high, the accuracy of variables capturing the presence of acute and chronic conditions is undoubtedly less.

**Patient Characteristics.** Patient, cancer, and treatment characteristics included: age; sex; presence of selected chronic comorbidities (cardiovascular disease, diabetes, liver disease, lung disease, renal disease, osteoarthritis, rheumatoid disease, thyroid disorder); body weight/nutritional status (obesity, malnutrition); proxies for health status (hospice and/or SNF care) and physical function (use of hospital bed, supplemental oxygen, walking aid, wheelchair); use of immunosuppressive therapy; history of blood disorders (anemia, neutropenia), infection, recent surgery (Appendix F), hospitalization, chemotherapy, and radiation therapy; cancer and chemotherapy regimen; and calendar year of chemotherapy initiation.

Age was assessed as of the first day of the first cycle of chemotherapy in the course. All other characteristics (except for recent surgery) were assessed during the 12-month pre-chemotherapy period; recent surgery was assessed during the 90-day pre-chemotherapy period. Chronic comorbidities were identified on the basis of ≥1 diagnosis codes on inpatient claims, ≥2 diagnosis codes on outpatient claims (excluding those for laboratory services) on different days, ≥1 procedure codes, and ≥1 drug codes, as appropriate (Appendix G). Blood disorders and infections were identified on the basis of ≥1 diagnosis codes (on inpatient and/or outpatient claims) and ≥1 drug codes, as appropriate.

**APPENDIX A: STUDY DESIGN SCHEMA**

**APPENDIX B: PRIMARY CANCERS**

**APPENDIX C: CHEMOTHERAPY AGENTS**

**APPENDIX D: PRIMARY AND SECONDARY CANCERS**

**APPENDIX E: INFECTION**

**APPENDIX F: SURGERIES**

**APPENDIX G: COMORBID CONDITIONS**

**REFERENCES**

1. Weycker D, Li X, Figueredo J, et al. Risk of chemotherapy-induced febrile neutropenia in cancer patients receiving pegfilgrastim prophylaxis: does timing of administration matter? *Support Care Cancer* 24(5):2309-16, 2016
2. Weycker D, Li X, Edelsberg J, et al: Risk and Consequences of Chemotherapy-Induced Febrile Neutropenia in Patients with Metastatic Solid Tumors. J Oncol Pract 11(1):47-54, 2015
3. Langeberg W, Siozon CC, Page JH, et al: Use of pegfilgrastim primary prophylaxis and risk of infection, by chemotherapy cycle and regimen, among patients with breast cancer or non-Hodgkin’s lymphoma. Support Care Cancer 22(8):2167-75, 2014
4. Weycker D, Malin J, Barron R, et al: Comparative effectiveness of filgrastim, pegfilgrastim, and sargramostim as prophylaxis against hospitalization for neutropenic complications in cancer chemotherapy patients. Am J Clin Oncol DOI 10.1097/COC.0b013e31820dc075, 2011
